# The Significance of Small Quantities of Albumen and of Sugar in the Urine

**Published:** 1906-02-03

**Authors:** 


### THE SIGNIFICANCE OF SMALL QUANTITIES OF ALBUMEN AND OF SUGAR IN THE URINE.

The question discussed by Dr. Robert W. Burnet[1] in a recent address is the clinical and pathological meaning of small and occasional quantities of albumen and of sugar in the urine, as seen in young persons, mostly males, who otherwise appear to be in good health. In the first place Dr. Burnet protests against the use of the term "physiological albuminuria," and asserts that the presence of albumen in the urine must be deemed an abnormal, and therefore a pathological, fact. It may possibly depend in some instances on temporary disturbances of function, and may thus not be the index of structural change, but all the same it is outside the limit of the processes of health. Hence it ought never to be viewed lightly. If the albuminuria is unaccompanied by casts or other histological evidences of renal disease, and if there is no increased arterial tension, it is permissible to anticipate that there is mere functional and temporary disturbance, and that this will pass away under suitable treatment. As a matter of fact experience shows that the majority of the cases now under discussion do make satisfactory progress, and ultimately get rid of their albuminuria. On the other hand, some drift into organic disease. The treatment of these cases includes the avoidance of strain, over-anxiety, over-work, unsuitable clothing, exposure to chills, unwholesome food, and alcoholic excess. A sea voyage is often attended with excellent results, and the same is true of a change to a mild, equable climate. Short of this the patient must lead a quiet and simple life, avoiding excesses of all kinds, in the hope that stable equilibrium in the body processes may be re-established. In reference to life insurance, some authorities urge that all proposers with albuminuria should be refused; but this is too arbitrary in regard to the cases now in question. All other circumstances being satisfactory these lives, in the event of the albuminuria proving of but temporary duration, may be accepted, but at an increased rate. The significance of small quantities of sugar in the urine in the class of patients now under discussion is probably more serious than is albuminuria. Not that it necessarily implies organic disease, but it certainly indicates considerable disturbance "in the working out of the chemical equations in the system." It cannot be so lightly dismissed as the same fact in persons of middle-age, where occasional glycosuria can often be traced to excess in diet or in the use of alcohol. Even without the general symptoms of diabetes sugar in the urine of young persons is of anxious significance, and probably means a distinct tendency to the development of diabetes. The presence of both albumen and sugar in the urine indicates serious disturbance in the metabolic processes, and calls for relief from the nervous strain which the patient may have been undergoing; under favourable conditions, however, these patients may continue in at least fair average health for many years.

[1] Brit. Med. Jour., January 20, 1906.

